# An application of restricted mean survival time in a competing risks setting: comparing time to ART initiation by injection drug use

**DOI:** 10.1186/s12874-018-0484-z

**Published:** 2018-03-09

**Authors:** Keri L. Calkins, Chelsea E. Canan, Richard D. Moore, Catherine R. Lesko, Bryan Lau

**Affiliations:** 1 Department of Epidemiology, Johns Hopkins Bloomberg School of Public Health, 615 N Wolfe St, Baltimore, MD 21205 USA; 2 School of Medicine, Johns Hopkins University, Baltimore, MD USA

**Keywords:** Survival analysis, Competing risks, Restricted mean survival time, HIV, Injection drug use, Antiretroviral therapy

## Abstract

**Background:**

Restricted mean survival time (RMST) is an underutilized estimand in time-to-event analyses. Herein, we highlight its strengths by comparing time to (1) all-cause mortality and (2) initiation of antiretroviral therapy (ART) for HIV-infected persons who inject drugs (PWID) and persons who do not inject drugs.

**Methods:**

RMST to death was determined by integrating the Kaplan-Meier survival curve to 5 years of follow-up. To account for the competing risks of death and loss-to-clinic when estimating time to ART, we calculated RMST to ART initiation by estimating the area between the survival curve for ART initiation and the cumulative incidence curve for death or loss-to-clinic. We standardized all curves using inverse probability of exposure weights.

**Results:**

We followed 3044 HIV-positive, ART-naive persons from enrollment into the Johns Hopkins HIV Clinical Cohort from 1996 to 2014. PWID had a − 0.19 year (95% confidence interval (CI): − 0.29, − 0.10) difference in survival over 5 years of follow-up compared to persons who did not inject drugs. There was no difference between the two groups in time not on ART while alive and in clinic (RMST difference = 0.08, 95% CI: -0.10, 0.36).

**Conclusions:**

PWID have similar expected time to ART initiation after properly accounting for their greater risk of death and loss-to-clinic.

**Electronic supplementary material:**

The online version of this article (10.1186/s12874-018-0484-z) contains supplementary material, which is available to authorized users.

## Background

Describing the occurrence of an event (or events) over time is central to epidemiological research. There are several estimands that can be used to summarize the occurrence of an event, such as providing survival or risk estimands for specific periods of time (e.g. 1-year survival or 5-year risk). (Note: we are using the term “survival” here to denote an event-free state, where the event is not constrained to be death.) The occurrence of an event in two or more groups is often compared using a risk difference, risk ratio, or hazard ratio [1–7]. In this paper we highlight restricted mean survival time (RMST) as an alternative estimand for the analysis of time-to-event data. RMST has attractive properties, namely it: 1) does not require the assumption of proportional hazards; 2) can summarize the difference in survival when survival curves initially diverge and later converge; and 3) provides information about absolute risk. RMST is calculated by integrating the survival function from the origin to some time *t*, and is interpreted as the average survival time within that interval [8–10]. Despite its attractive properties, epidemiological studies utilizing RMST remain rare [11–14], particularly in an observational setting (which would require adjustment of RMST for potential confounding), or in the presence of competing risks (events that preclude the event of interest from occurring).

The first objective of this paper is to promote the use of the RMST in the epidemiology and medical literature by briefly reviewing its calculation, interpretation, strengths, and limitations. The second objective is to detail an approach to standardize the RMST to account for confounding in the setting of competing events using inverse probability weighting. We illustrate estimation of the adjusted RMST to describe time to death in an HIV clinical cohort among persons with and without a history of injection drug use (IDU) as a risk factor for HIV acquisition. We further illustrate use of RMST to compare time to initiation of antiretroviral therapy (ART) among persons who inject drugs (PWID) and persons who did not inject drugs, properly accounting for persons who experienced a competing event (death or loss-to-clinic).

## Methods

The definite integral of the survival curve from the time origin to some specified follow-up time *t*, where *t* is less than or equal to the maximum observed follow-up time, provides an estimate of the expected event-free time from 0 to *t* [15]. The area under the survival curve, *A*(*t*) is also known as the RMST. This is in contrast to the marginal expected survival time, which is only estimable (without extrapolation) when the survival curve goes to zero during the observation time [16]. The “restricted” component of the mean survival calculation avoids extrapolating the integration beyond the last observed time point. A particular strength of RMST is the ease of interpretation. The difference in the area under two survival curves *A*_1_(*t*) – *A*_2_(*t*) can be interpreted as the extension (or contraction) of the expected (mean) survival time by time *t* associated with membership in group 1 or with treatment 1, as compared to group 2 or treatment 2. One could easily take the ratio rather than the difference.

### Statistical methods

RMST, which was originally proposed by Irwin [8] and later adapted by Kaplan and Meier [17] is defined by [18]:

1 $$ A(t)=E\left[\min \left(T,{t}^{\ast}\right)\right]={\int}_0^{t^{\ast }}S(t) dt $$

where *T* is the time to the event of interest, *t*^∗^ is the point in time to which RMST is being calculated between 0 and the maximum follow-up, and *S*(*t*) is the survival function over time *t*. *S*(*t*) can be estimated using a variety of methods, i.e. non-parametrically, semi-parametrically, or parametrically. We follow convention and represent random variables with capital letters and possible realizations of those random variables with lowercase letters. Challenges in estimating RMST include: 1) deciding how to estimate the function *S*(*t*); 2) adjusting for confounders; and 3) estimating the integral ${\int}_0^{t^{\ast }}S(t) dt$.

We focus on the estimation of *S*(*t*) using a step function, specifically, the Kaplan-Meier survival function. The use of a step function allows for a simple integration of the survival function to time *t* by summing the area under each rectangular step of the survival curve (i.e., a Reimann sum) [10]:

2 $$ \widehat{A}(t)={\sum}_k\widehat{S}\left({t}_{k-1}\left)\right({t}_k-{t}_{k-1}\right) $$

where *t*_*k*_ are the ordered event times observed over (0, *t*^∗^] and *t*^∗^ is included in the set of *t*_*k*_*, t*_0_ = 0, $\widehat{S}(t)$ is the survival estimate, and by definition $\widehat{S}\left({t}_0\right)=1$. We can interpret $\widehat{A}(t)$ as the restricted mean time spent event-free through time *t*. Conversely, the integral over (0, *t*^∗^] of the complement of the survival function, *F*(*t*) = 1 − *S*(*t*), which is the cumulative incidence function (CIF) is the expected time after having the event through time *t*^∗^ [19].

There are several options for generating covariate-adjusted curves to account for confounding or non-random censoring [20]. Here we detail how to obtain standardized survival curves using inverse probability weighting. Inverse probability weighting has several attractive properties compared to other methods for generating covariate-adjusted curves, namely that it produces survival curves that are marginalized over the distribution of covariates in the study sample rather than requiring a covariate profile be specified and is straightforward to implement [21, 22]. Inverse probability weighting is an extension of direct standardization [23] and it has been shown that a weighted Kaplan-Meier estimator can provide an unbiased estimate for an adjusted survival curve using an inverse probability weighted hazard function [22]. We define an individual’s weight as ${\widehat{W}}_i=\widehat{P\left({X}_i\right)}/\widehat{P\left({X}_i|{Z}_i\right)}$, where *Z* is set of covariates sufficient to satisfy the assumption of conditional exchangeability between two groups, *X* = 1 and *X* = 0. We suggest incorporating causal knowledge and relying on causal diagrams to identify a sufficient set of covariates to satisfy this assumption [24]. Note that we stabilized the weights by the marginal probability of exposure, but non-stabilized weights are also possible. Other assumptions are necessary to interpret contrasts between RMST for *X* = *x* causally, but a full discussion of causal inference is beyond scope of this paper; see [23, 25, 26] for more details. Briefly, one sufficient set of assumptions includes, in addition to assuming conditional exchangeability between the exposed and unexposed: 1) positivity (everyone has a non-zero probability of being exposed) [27, 28]; 2) treatment version irrelevance [29–31]; 3) no interference (one person’s exposure does not affect another’s outcome) [32, 33]; 4) no measurement error [26, 34]; and 5) correct model(s) specification.

The RMST can also be used in settings where there is confounding and competing events. When a competing event precludes the event of interest from occurring, an alternate estimator is recommended. With *j* = 1, …, *J* different events, the CIF is the joint probability that an event occurs and the event is of type *j*, *F*_*j*_(*t*) = *P*(*T* < *t*, *J* = *j*). The inverse probability weighted CIF for event type *j* can be estimated [6]:

3 $$ {\widehat{F}}_j(t)=\sum \limits_{t_k\le t}\left[{\widehat{S}}^{\widehat{W}(t)}\left({t}_{k-1}\right)\right]\left[\frac{d_j^{\widehat{W}(t)}\left({t}_k\right)}{n^{\widehat{W}(t)}\left({t}_k\right)}\right] $$

where the overall inverse probability weighted survival function is ${\widehat{S}}^{\widehat{W}(t)}(t)=\mathit{\exp}\left\{-\sum \limits_{t_k\le t}{d}^{\widehat{W}(t)}\left({t}_k\right)/{n}^{\widehat{W}(t)}\left({t}_k\right)\right\}$, ${d}_j^{\widehat{W}(t)}$and ${n}^{\widehat{W}(t)}\left({t}_k\right)$ are the inverse probability weighted number of events of type *j* and number of individuals at risk, respectively, at time *t* [6]. $\frac{d_j^{\widehat{W}(t)}\left({t}_k\right)}{n^{\widehat{W}(t)}\left({t}_k\right)}$ is the cause-specific hazard ratio at time *t*_*k*_. The complement of the CIF provides the survival function for the *j*th event, *S*_*j*_(*t*) = 1 − *F*_*j*_(*t*). The lowest value ${\widehat{S}}_j(t)$ may attain is bounded by the sum of the cumulative incidences of the competing events, i.e., ${\sum}_{J\ne j}{\widehat{F}}_J(t)$, since ${\widehat{F}}_j(t)+\sum \limits_{J\ne j}{\widehat{F}}_J(t)+{\widehat{S}}^{\widehat{W}(t)}(t)=1$.

Integration of *F*_*j*_(*t*) or *S*_*j*_(*t*) over [0, *t*^∗^] provides the expected time after the occurrence of event *j* through *t*^∗^ and the expected time prior to the occurrence of event *j* through *t*^∗^, respectively. Note the interpretation of these integrals remains the same even if an individual experiences a competing event prior to *t*^∗^. For example, if the event of interest *j* is initiation of ART and the competing event is death, then the integration of *S*_*j*_(*t*) over [0, *t*^∗^] would be interpreted as the expected time prior to ART through *t*^∗^, even if the individual died before initiating ART. Similarly, the integration of *F*_*j*_(*t*) over [0, *t*^∗^] would be interpreted as the expected time after ART initiation through *t*^∗^ even if the individual dies following ART initiation but prior to *t*^∗^. The restricted mean lifetime spent in a state free of all events, including the event of interest and competing events, can be calculated by taking the difference in the integration of the complement of the CIF from the *j*th event and the integration of the CIF of all other events. Therefore this estimand is:

4 $$ {\int}_0^{t^{\ast }}{S}_{J=j}(t) dt-{\int}_0^{t^{\ast }}{\sum}_{J\ne j}{F}_J(t) dt={A}_{J=j}(t)-{L}_{J\ne j}(t) $$

where ${A}_{J=j}(t)={\int}_0^{t^{\ast }}1-{F}_{J=j}(t) dt$ is the expected survival time to the *j*th event and ${L}_{J\ne j}(t)={\int}_0^{t^{\ast }}{F}_{J\ne j}(t) dt$ is the expected time spent in all non-*j* events (i.e. the competing events). That is, the area under the CIF for event *j* is the expected time after having event *j*, therefore the integral of the complement, *A*_*J* = *j*_(*t*), corresponds to the expected time remaining in a state free of the *j*th event. However, this includes being in a non-*j* event state. Because we are interested in time spent free of all events, the expected time spent in the non-*j* events states needs to be removed and is provided in Eq. 4. *F*_*J* = *j*_(*t*) and *F*_*J* ≠ *j*_(*t*) can be estimated using Eq. 3, where the event type can either be the event of interest, *j* or the composite of the competing events.

### Application

Despite demonstrated success of combination ART on reduced morbidity, mortality, and HIV transmission risk [35, 36], many populations experience significant barriers to and delays in the initiation of ART. PWID consistently experience delayed treatment and lower rates of viral suppression [37–41]. To demonstrate how RMST can provide further insight into these disparities, we describe 1) time to all-cause mortality and 2) time to ART initiation among a cohort of persons engaged in HIV clinical care, stratifying by report of IDU as an HIV acquisition risk factor. We begin by examining all-cause mortality because we anticipate mortality to be an important competing event for ART initiation and to serve as an example of the implementation of the use of inverse probability-weighted RMST in a setting with no competing events.

We followed individuals who enrolled in continuity HIV care at the Johns Hopkins Moore Clinic for HIV Care from 1994 to 2014, who consented to share their medical record data with the Johns Hopkins HIV Clinical Cohort (> 90% of patients) and who had not yet initiated ART. When analyzing time to all-cause mortality, patients were followed from cohort enrollment until death, 5 years of follow-up or administrative censoring in June 2014. Date of death was ascertained through periodic matches against the National Death Index and the Social Security Death Index, so patients would not have to return to clinic in order to have their date of death measured and there are no competing events. While ART initiation impacts all-cause mortality, it is on the causal pathway between injection drug use and mortality; because this analysis focuses on the total effect of history of injection drug use on mortality, we do not account for ART.

When analyzing time to ART initiation, patients were followed from clinic enrollment until ART initiation (defined as the initiation of a three-drug regimen on a single day), loss-to-clinic (defined as the date on which a patient has gone one year without a CD4 or HIV RNA measurement or a clinic visit), death, or 5 years of follow-up or administrative censoring in June 2014. Patients must be under clinical care in order to receive treatment. In this analysis, we assume that patients only receive ART through our clinic. Therefore, patients must return to clinic for the outcome to occur. By definition, we can never observe ART initiation among patients who die or drop out of clinical care (loss-to-clinic) prior to ART initiation; thus we consider death and loss-to-clinic to be competing events in this analysis.

We excluded 285 individuals (8.6%) with missing covariate information (33 missing both CD4 count and HIV RNA level, 18 missing CD4, 234 missing HIV RNA). The final analytic sample contained 3044 patients.

We generated stabilized inverse probability of exposure weights to standardize the Kaplan-Meier survival curves for all analyses. The denominators of the weights were estimated using predicted probabilities from a logistic regression model for IDU as an HIV acquisition risk factor conditional on the following baseline characteristics: sex, race, prior AIDS diagnosis, prior mono- or dual-antiretroviral therapy, age, CD4 cell count and log_10_ HIV RNA level. The numerators of the weights were the marginal probability of being in the exposure group that was observed for that individual. Baseline covariate values were those measured closest to enrollment date within a window of 6 months before to 6 months after enrollment and prior to ART initiation. Continuous covariates were modeled with basis cubic splines [42].

When analyzing time to ART initiation we generated two sets of curves for PWID and persons who did not inject drugs: 1) the inverse probability weighted CIF for time to ART initiation based on Eq. 3 (weights were as defined above); and 2) the inverse probability weighted cause-specific CIF for a composite event defined as death or loss-to-clinic. Because death and loss-to-clinic preclude the occurrence and/or observation of ART initiation, we treat these two events as competing events [43]. We present the expected time not on ART while alive and enrolled in the clinic which is, ${\widehat{A}}_{J= ART\  initiation}(t)-{\widehat{L}}_{J\ne ART\  initiation}(t)$, where ${\widehat{A}}_{J= ART\  initiation}(t)$ is the area under the survival curve for time to ART initiation and ${\widehat{L}}_{J\ne ART\  initiation}(t)$ is the area under the CIF for death or loss-to-clinic prior to ART initiation.

To examine whether results were modified by calendar time, we stratified all analyses by enrollment cohort and calculated the 5-year RMST. Enrollment cohorts were loosely defined by changes to ART initiation guidelines (1996–2001, 2002–2007, 2008–2014). We selected 5 years as the time point in which to calculate the RMST (t*) because of our stratified analysis by enrollment cohort. The most recent enrollment cohorts would have a maximum 5.5 years of follow-up prior to administrative censoring in June 2014. Furthermore, the majority of ART initiation events are likely to occur within 5 years of clinical enrollment, so RMST differences in ART initiation are unlikely to change significantly after 5 years. For all estimates, we calculated 95% confidence intervals (CI) using the 2.5th and 97.5th percentiles of 10,000 non-parametric bootstrap estimates based on unrestricted random samples from the original data [44]. All analyses were conducted using R version 3.3.1 [45]. Sample R code for calculating RMST with competing risks is provided in Additional file 1.

## Results

Of the 3044 patients included in the time to ART analysis a majority were male (65.2%) and non-Hispanic Black (77.2%). At clinic enrollment, the median age was 39 years (interquartile range (IQR) = 33–45 years), 22.1% of patients had a prior AIDS diagnosis and 25.9% had received prior mono- or dual-antiretroviral therapy. The baseline median CD4 cell count was 279 (IQR = 94–480) cells/μL and the baseline median log_10_ HIV RNA was 4.6 (IQR = 3.9–5.3) copies/mL. Overall, 1155 (37.9%) of the patients were PWID. PWID were older, more likely to be black, more likely to have received prior mono-or dual-antiretroviral therapy, and more likely to enroll in care prior to 2002 (Table 1).

**Table 1:** Patient baseline characteristics by history of injection drug use

	N (%)		
	Persons who did not inject drugs *N* = 1889 (62.1%)	PWID *N* = 1155 (37.9%)	Total *N* = 3044
Age, median (IQR)	38.2 (31.4–45.3)	41.2 (36.7–46.4)	39.5 (33.7–45.8)
Sex			
Female	655 (34.7)	403 (34.9)	1058 (34.8)
Male	1234 (65.3)	752 (65.1)	1986 (65.2)
Race			
Black	1395 (73.8)	955 (82.7)	2350 (77.2)
White	415 (22.0)	184 (15.9)	599 (19.7)
Other	79 (4.2)	16 (1.4)	95 (3.1)
CD4, median (IQR)	280 (89–478)	279 (103–485)	279.5 (94–480)
Log_10_ RNA, median, (IQR)	4.7 (3.9–5.3)	4.6 (3.8–5.2)	4.6 (3.9–5.2)
Prior AIDS			
Yes	411 (21.8)	261 (22.6)	672 (22.1)
No	1478 (78.2)	894 (77.4)	2372 (77.9)
Prior ART			
Yes	431 (22.8)	357 (30.9)	788 (25.9)
No	1458 (77.2)	798 (69.1)	2256 (74.1)
Year of clinic enrollment			
1996–2001	971 (51.4)	787 (68.1)	1758 (57.8)
2002–2007	468 (24.8)	222 (19.2)	690 (22.7)
2008–2014	450 (23.8)	146 (12.6)	596 (19.6)

Abbreviations: *ART*, antiretroviral therapy, *IQR* interquartile range, *PWID*, persons who inject drugs

**Fig. 1:**
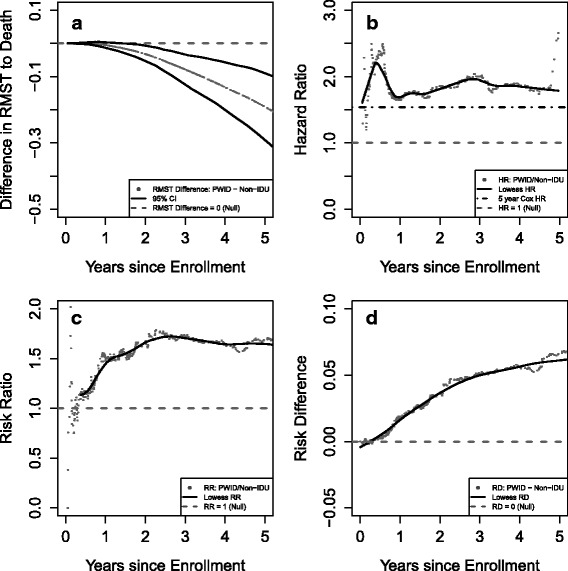
The effect of follow-up time on four different effect estimates comparing all-cause mortality by IDU. Panel **a**) displays the standardized^a^ difference (with 95% confidence interval) in restricted mean time to all-cause mortality by history of injection drug use over follow-up time. Panel **b**) displays the standardized^a^ all-cause hazard ratio (HR) over follow-up time. Panel **c**) displays the standardized^a^ all-cause mortality risk ratio (RR) over follow-up time. Panel **d**) displays the standardized^a^ all-cause mortality risk difference (RD) over follow-up time. ^a^ Curves are standardized to the distribution of sex, race, AIDS diagnosis, prior ART mono- or dual-therapy, age, CD4 cell count, log_10_ HIV viral load, Hepatitis C, history of smoking, and hazardous alcohol use in the total sample at clinic enrollment using inverse probability of exposure weights. ^b^ Dashed grey lines represent the null hypothesis (i.e. difference in RMST = 0, HR = 1, RR = 1, and RD = 0) in each panel. ^c^ The dot-dash black line on panel **b** is the HR estimated from the Cox Proportional Hazards Model. ^d^ The solid black line on panels **b**, **c**, and **d** is the lowess smoother to show the overall trend of the estimands. ^e^ The RR and RD is defined as $\left(1-{S}_{PWID}^w(t)\right)/\left(1-{S}_{non- PWID}^w(t)\right)$ and $\left(1-{S}_{PWID}^w(t)\right)-\left(1-{S}_{non- PWID}^w(t)\right)$, respectively, where ${S}_{\bullet}^w(t)$ is the weighted survival curve

All-cause mortality was higher among PWID compared to persons who did not inject drugs, emphasizing the importance of accounting for death as a competing risk when examining time to ART initiation. The 5-year RMST to death was 4.51 years for PWID and 4.70 years for people who did not inject drugs. In other words, in the first 5 years following clinic enrollment, PWID spend an average of 4.51 years alive and people who do not inject drugs spend an average of 4.70 years alive. The difference in restricted mean survival between PWID and people who did not inject drugs was − 0.19 years (95% CI: -0.29, − 0.09). Panel A of Fig. 1 shows the difference in RMST to all-cause mortality comparing PWID and persons who did not inject drugs between 0 and 5 years after clinic enrollment. There is a near zero difference in expected time to death between PWID and persons who did not inject drugs until approximately 2 years after clinic enrollment. Following two years of follow-up, PWID begin to experience a shorter time to all-cause mortality than people who did not inject drugs, resulting in an increasingly negative difference in RMST over time.

Figure 1 shows the relationship between PWID and all cause mortality for the RMST as well as three of the most common estimands: the hazard ratio (HR), the risk ratio (RR), and the risk difference (RD). The RR and RD were calculated using the Kaplan-Meier estimates for the survival function. As stated above, PWID experience an increasingly shorter time to death after 2 years of clinic enrollment based on the RMST. The HR is a more variable measure but for the majority of time points at which an event occurs, the hazard of all-cause mortality is higher among PWID and the HR estimated from the Cox model is 1.54 (Fig. 1, panel B). The RR is approximately 1 (Fig. 1, panel C) and the RD is approximately 0 (Fig. 1, panel D) for a few months after enrollment before increasing. Overall the four different effect estimates indicate an increased all-cause mortality for PWID compared to patients who do not inject drugs.

When we examine all-cause mortality by era of clinic enrollment (Table 2), the difference in time to death comparing PWID to persons who did not inject drugs in the first five years after clinic enrollment is larger in magnitude in the more recent time periods. The difference in expected time to death over 5 years of follow-up among PWID compared to persons who did not inject drugs is − 0.15 years (95% CI: -0.27, − 0.02) for those entering care between 1996 and 2001, − 0.35 years (95% CI: -0.64, -0.05) for those entering care between 2002 and 2007, and − 0.30 years (95% CI: -0.53, 0.01) for those entering care between 2008 and 2014.

**Table 2:** Standardized^a^ 5-year restricted mean time to all-cause mortality by history of injection drug use

	Expected time (years) to All-Cause Mortality					
	PWID		Persons who did not inject drugs		Difference	
	RMST	95% CI^b^	RMST	95% CI	RMST	95% CI
All years	4.51	4.42, 4.57	4.70	4.65, 4.74	−0.19	−0.29, −0.09
1996–2001	4.47	4.36, 4.58	4.62	4.54, 4.68	−0.15	−0.27, − 0.02
2002–2007	4.45	4.18, 4.71	4.80	4.56, 5.00	−0.35	−0.64, − 0.05
2008–2014	4.58	4.36, 4.87	4.88	4.80, 4.97	−0.30	−0.53, 0.01

^a^Estimates are standardized to the distribution of sex, race, AIDS diagnosis, prior ART mono- or dual-therapy, age, CD4 cell count, and log_10_ HIV viral load in the total sample at clinic enrollment using inverse probability of exposure weights

^b^95% CI was based upon the 2.5th and 97.5th percentiles of 10,000 non-parametric bootstrap resamples

**Fig. 2:**
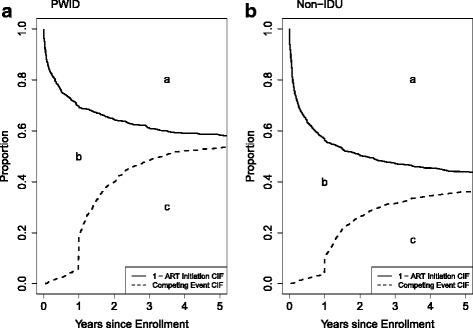
Standardized^a^ cumulative incidence functions (CIF)^b^ for ART initiation and for the composite competing event, death or loss-to-clinic, for PWID (**a**) and non-IDU (**b**). ^a^ Curves are standardized to the distribution of sex, race, AIDS diagnosis, prior ART mono- or dual-therapy, age, CD4 cell count and log_10_ HIV RNA level in the total sample at enrollment using inverse probability of exposure weights. ^b^Solid line is the standardized 1-CIF (cumulative incidence function) for ART initiation. Dashed line is the CIF for the composite competing event, death or loss-to-clinic. Area “a” is the 5-year restricted mean time after ART initiation; “b” is the 5-year restricted mean time spent not on ART while alive and in clinic; “c” is the 5-year restricted mean time after death or loss-to-clinic

Figure 2 shows the CIF for death or loss-to-clinic and the complement of the CIF for ART initiation by history of IDU. These curves depict the restricted mean time after ART initiation (Fig. 2, area ‘a’) and the restricted mean time after the competing events of mortality and loss-to-clinic (Fig. 2, area ‘c’). Area ‘b’, the 5-year restricted mean time spent not on ART while alive and retained in the clinic was 1.51 years (95% CI: 1.44, 1.87) for PWID and 1.43 years (95% CI: 1.37, 1.64) for persons who did not inject drugs. PWID experienced a 0.08 year delay (95% CI: -0.10, 0.38) in time to ART initiation compared to persons who did not inject drugs. There was a measurable difference in time spent not on ART while alive and enrolled in clinic for those entering care between 2008 and 2014; the 5-year difference in restricted mean time to ART while alive and in clinic was 1.39 years (95% CI: 0.15, 1.98), representing a delay in initiation of ART for PWID as compared to those who did not inject drugs (Table 3).

**Table 3:** Standardized^a^ 5-year RMST^b^ spent not on ART but alive and retained in clinic^b^ by IDU

	Expected time (years) to ART initiation					
	PWID		Persons who did not inject drugs		Difference	
	RMST	95% CI^c^	RMST	95% CI	RMST	95% CI
All years	1.51	1.44, 1.87	1.43	1.37, 1.64	0.08	−0.10, 0.38
1996–2001	1.61	1.51, 2.25	1.54	1.48, 1.91	0.07	−0.26, 0.67
2002–2007	1.48	1.25, 2.32	1.61	1.26, 2.09	−0.13	−0.82, 0.72
2008–2014	2.87	2.66, 3.52	1.48	1.34, 2.73	1.39	0.15, 1.98

^a^Estimates are standardized to the distribution of sex, race, AIDS diagnosis, prior ART mono- or dual-therapy, age, CD4 cell count and log_10_ HIV viral load in the total sample at clinic enrollment using inverse probability of exposure weights

^b^The RMST spent not on ART but Alive and Retained in Clinic refers to the difference of the integration of the 1-CIF for ART initiation minus the CIF for the composite of mortality and loss-to-clinic

^c^Based on integration between 1-CIF for ART 2.5th and 97.5th percentiles of 10,000 non-parametric bootstrap resamples

**Fig. 3:**
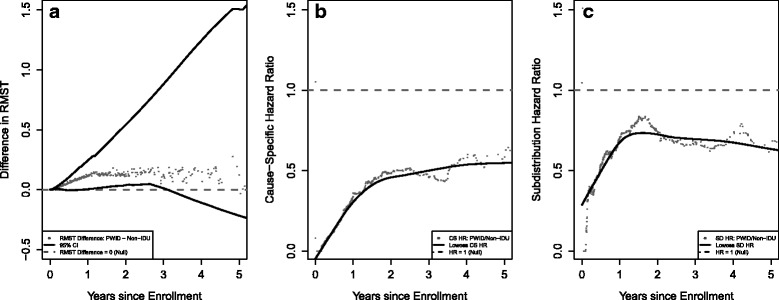
The effect of follow-up time on three different effect estimates comparing ART initiation by IDU accounting for the competing event of death and loss-to-clinic. Panel **a**) displays the standardized^a^ difference (with 95% confidence interval) in restricted mean time in years spent not on ART while alive and in clinic by history of injection drug use over follow-up time. Panel **b**) displays the standardized^a^ cause-specific hazard ratio (HR) over follow-up time. Panel **c**) displays the standardized^a^ subdistribution mortality risk ratio (RR) over follow-up time. ^a^ Curves are standardized to the distribution of sex, race, AIDS diagnosis, prior ART mono- or dual-therapy, age, CD4 cell count, log_10_ HIV viral load, Hepatitis C, history of smoking, and hazardous alcohol use in the total sample at clinic enrollment using inverse probability of exposure weights. ^b^ Dashed grey lines represent the null hypothesis (i.e. difference in RMST = 0 and HR = 1) in each panel. ^c^ The solid black line on panels B and C is the lowess smoother to show the overall trend of the estimands

We compare the RMST spent not on ART but alive and in clinic to the cause-specific and subdistribution hazard ratios where ART initiation is the outcome of interest and death or loss-to-clinic is the competing event in Fig. 3. We see that PWID spend a longer time alive and in clinic while not on ART as compared to persons who do not inject drugs, and this difference becomes less pronounced as t* increases (Fig. 3, panel A). Both the cause-specific (Fig. 3, panel B) and the subdistribution hazard ratios (Fig. 3, panel C) are variable early into follow-up but level off and are consistently below 1. The subdistribution hazard ratio is closer to the null than the cause-specific. The inference from both the cause-specific and the subdistribution hazard ratio is that PWID have a lower rate (i.e., cause-specific hazard) and have a lower qualitative risk (i.e., subdistribution hazard) of ART initiation compared to persons who do not inject drugs.

## Discussion

Using the restricted mean survival time approach, we evaluated the expected time to all-cause mortality and ART initiation by history of IDU among a cohort of HIV-infected patients receiving treatment in Baltimore, MD. We estimated that, between 1996 and 2014, PWID had an expected 5-year restricted mean survival that was shorter than people who did not inject drugs by 0.19 years, after standardizing on baseline clinical and demographic covariates. We did not see a difference in time to all-cause mortality after 5 years of follow-up in each era of clinic enrollment, likely due to the relatively few events occurring within 5 years of clinic enrollment. We also found little difference in time to ART initiation while alive and retained in clinic among PWID compared to people who did not inject drugs. Other studies that have reported disparities in time to ART initiation among PWID compared to persons who did not inject drugs may not have accounted for death or loss-to-clinic as competing risks explicitly.

This study had several strengths. The sample size was sufficiently large to examine trends in time to ART initiation stratified by enrollment cohort, which is of particular interest because of changing treatment guidelines. We highlighted the novel approach of using inverse probability weighting to standardize the RMST in the setting of competing events. By using inverse probability weights to standardize cohorts of PWID and persons who did not inject drugs to have the same distribution of baseline covariates, we were able to estimate restricted mean times to ART initiation that are not confounded by different clinical indications for treatment.

This study also had limitations. Data were collected from a single HIV clinic in an urban academic center and may not be generalizable to other sites. Additionally, we were unable to ascertain whether patients who were lost-to-clinic began treatment at a different clinic. As such, our estimates of time to ART initiation may be biased. Finally, we examined differences in RMST to ART initiation by self-reported history of IDU as an HIV acquisition risk factor. History of IDU may be under-reported due to social desirability bias. Furthermore, history of IDU does not correlate perfectly with ongoing IDU and our estimates should not be interpreted as such.

Restricted mean survival time is a useful alternative to the traditional hazard ratio. The use of proportional hazards models in the presence of competing risks whether examining the cause-specific or subdistribution HR remains subject to the proportionality assumption. Relaxing the proportional hazard assumption is possible, but doing so means no longer having a single summary estimate of association [15]. The RMST provides a single summary measure of survival through time *t*^∗^ that avoids this pitfall. Furthermore, the cause-specific HR may not translate to an actual change in risk [43], while the RMST is estimated directly from risk functions. Nevertheless, the hazard ratio, relative risk and risk difference remain important measures in their own right [6].

As was demonstrated in the comparison of the RMST, HR, RR, and RD, all four measures are useful in describing the difference in all-cause mortality by history of injection drug use. The RD can highlight the public health importance of a particular exposure of interest as it is measured on an absolute scale and the HR provides the instantaneous relative rate at a particular point in time. Compared to the HR, an advantage of RMST is that it has more power to detect differences between exposure groups when the HR is close to 1 [18]. Further, unlike the HR, RR, and RD that compare exposure groups at a single point in time, the comparison of RMST between exposure levels summarizes the difference in expected mean time to an event for a given time interval. This is especially useful when comparing survival functions that diverge and later converge or cross. A final advantage of the RMST is the ease of interpretability when summarizing delays in care or decreases in survival. The use of RMST in our analysis to describe the difference in time not on ART while alive and in clinic facilitated the interpretation of our results by providing a clinically meaningful measure.

## Conclusions

RMST can be calculated easily using non-parametric and semi-parametric estimators. We have demonstrated use of RMST to determine that PWID have lower expected survival over the first five years of clinical engagement compared to persons who did not inject drugs. However, PWID have similar expected time to ART initiation after properly accounting for their greater risk of death and loss-to-clinic as competing events. In conclusion, the restricted mean survival time is a useful alternative in analyzing time-to-event data that can provide supplementary information to traditional survival estimands (e.g. the hazards ratio or risk difference).

## Additional file

Additional file 1: Contains the R code used to set up the analysis, as well as an outline of how the various tables in results section were generated. (DOCX 94 kb)

## Abbreviations

ART: Antiretroviral therapy
CI: Confidence Interval
CIF: Cumulative Incidence Function
HR: Hazard ratio
IDU: Injection drug use
IQR: Interquartile range
PWID: Persons who inject drugs
RD: Risk difference
RMST: Restricted Mean Survival Time
RR: Risk ratio
