# Supplementary material for: An application of restricted mean survival time in a competing risks setting: comparing time to ART initiation by injection drug use
Source: BMC Med Res Methodol. 2018 Mar 9;18:27. doi: 10.1186/s12874-018-0484-z (PMC5845164; doi:10.1186/s12874-018-0484-z)
Supplement: Supplementary file 1 — Contains the R code used to set up the analysis, as well as an outline of how the various tables in results section were generated. (DOCX 94 kb) [file 12874_2018_484_MOESM1_ESM.docx]

**Appendix:**

# R code for calculating the restricted mean survival time to initiation of

# ART in standard survival analysis (Table 2) and competing risk settings

# (Table 3)

library(survival)

library(car)

library(splines)

library(sfsmisc)

###########################################################################

###########################################################################

################

### TABLE 2 ####

################

# R code for calculating the restricted mean survival time to all-cause mortality

#############################################################################

## Step 1: Create stabilized inverse probability weights

# Fit logistic regression model for IDU status based on age, sex, race,

# cd4, log HIV viral load, prior AIDS diagnosis, and prior ART

# Stabilize the weights by the conditional probability of exposure, use the

# stabilized weights for all KM curves

ipw<-predict(glm(idu~bs(agein)+male+factor(race)+bs(cd4_baseline)+ bs(logvl_baseline)+aids0+priorart+factor(newera), data=dat,family=binomial),type="response")

s.ipw<-(dat$idu*(mean(dat$idu)/(ipw)))+(1-dat$idu)*

((1-mean(dat$idu))/(1-ipw))

###########################################################################

## Step 2: Fit a weighted KM curve for the event of interest

AUCsurvival<-Surv(dat$start,dat$end,dat$death)

AUCfit_adj<-survfit(AUCsurvival~idu,weights=s.ipw,data=dat,

type="kaplan-meier")

###########################################################################

## Step 3: Save the event times and survival at each event time

# identify times for all events

t.events <- data.frame(AUCfit_adj$time[AUCfit_adj$n.event>0])

names(t.events)<-"t.events"

# Note that event times are stored in order of exposure.

# All unexposed are stored first, then all exposed

# Obtain the time difference between each event time

t.events$diff<-c(NA,diff(t.events$t.events))

# Event times will be ascending until they restart for the exposed group

# flags the row where event times restart (i.e. moving from unexposed to

# exposed times)

first.exp<-which(t.events$diff<0)

# flags the row of the last event time for the unexposed group

last.unexp<-which(t.events$diff<0)-1

# total number of event times

total.length<-length(t.events$t.events)

#create vector of event times for unexposed group

# add 0 as the first entry in the event time vector for unexposed group

t.unexp <- t.events[1:last.unexp,1]

t.unexp<-append(0,t.unexp)

# again for exposed

t.exp <- t.events[first.exp:total.length,1]

t.exp<-append(0,t.exp)

# survival curve at all of the event times

s.events <- AUCfit_adj$surv[AUCfit_adj$n.event>0]

#create vector of survival rates for unexposed group

# and add 1 as the first entry in the survival vector for unexposed group

s.unexp <- s.events[1:last.unexp]

s.unexp<-append(1,s.unexp)

# again for exposed group

s.exp <- s.events[first.exp:total.length]

s.exp<-append(1,s.exp)

###########################################################################

## Step 4: Obtain values for Table 2: time to ART initiation

#set maxiumum follow-up time to 5 years (1826.25 days)

tmax=1826.25

#find maximum event time for unexposed and exposed groups that is

# less than 5 years

t.un<-t.unexp[which(t.unexp<=tmax)]

t.ex<-t.exp[which(t.exp<=tmax)]

#find number of event times in the unexposed and exposed groups

tmax.unexp<-length(t.un)

tmax.exp<-length(t.ex)

# As the Kaplan-Meier is a step function the restricted mean survival time

# for initiation of ART may be calculated by determining the rectangle

# area under each step of the survival curve (i.e., width of the step

# times the height) and then iteratively adding these rectangles

area.unexp <- NULL

for (i in 1:tmax.unexp) {

area.unexp[i] = s.unexp[i-1]*(t.unexp[i]-t.unexp[i-1])

}

# sum each of the rectangles up to last event time <5 years

# then add on the last area between last event time and 5 years

AUC_unexp<-sum(area.unexp)+s.unexp[tmax.unexp]*

(tmax-t.unexp[tmax.unexp])

#Calculate RMST for exposed group by iteratively adding the area under each step of the survival function

area.exp <- NULL

for (i in 1:tmax.exp){

area.exp[i] = s.exp[i-1]*(t.exp[i]-t.exp[i-1])

}

AUC_exp<-sum(area.exp)+s.exp[tmax.exp]*(tmax-t.exp[tmax.exp])

#Calculate the difference and ratio of RMST between exposed and unexposed groups for Table 2

AUC_diff<-AUC_exp-AUC_unexp

AUC_ratio<-AUC_exp/AUC_unexp

###########################################################################

###########################################################################

################

### TABLE 3 ####

################

# R code for calculating the restricted mean survival time to initiation of

# effective antiretroviral therapy in non-competing risk setting

# Note this is essentially using the survfit command weighted by inverse

# probability weights for (1) composite event, 2) event of interest, and

# 3) competing event. From (1) the overall survival function is obtained.

# From (2) and (3) the survfit command is only being used to obtain the

# weighted number of events and risk set at failure time. Then using the

# overall survival function, event failure times of event of interest,

# weighted events of interest, and weighted risk set, the cumulative

# incidence function can be calculated using equation 4 in main text

###########################################################################

## Step 1: Create a composite outcome equal to any of the following: ART

## initiation, death, loss-to-clinic (administrative censoring after 1

## year)

dat$composite <- ifelse(dat$haart==1,1,

ifelse(dat$death==1,1,

ifelse(dat$enddt<max(dat$enddt)-365.25,1,0)))

###########################################################################

## Step 2: Fit a weighted KM curve to the composite outcome

AUCsurvival_composite <- Surv(dat$start,dat$end,dat$composite)

AUCfit_adj_composite<-survfit(AUCsurvival_composite~idu,weights=s.ipw,

data=dat,type="kaplan-meier")

###########################################################################

## Step 3: Save the survival estimates across all points in time from the

## composite survival function, then create a lag

t.events.composite <-

data.frame(AUCfit_adj_composite$time[AUCfit_adj_composite$n.event>0])

#identify time between all failure time points

names(t.events.composite)<-"t.events.composite"

t.events.composite$diff<-c(NA,diff(t.events.composite$t.events.composite))

#subtract each event time from the subsequent event time to identify the row # at which event times switch from the unexposed group to the exposed
# group

last.unexp.composite<-which(t.events.composite$diff<0)-1

first.exp.composite<-which(t.events.composite$diff<0)

total.length.composite<-length(t.events.composite$t.events.composite)

#create vector of event times for unexposed and then exposed groups

t.unexp.composite <- t.events.composite[1:last.unexp.composite,1]

t.exp.composite <-

t.events.composite[first.exp.composite:total.length.composite,1]

s.events.composite <-

AUCfit_adj_composite$surv[AUCfit_adj_composite$n.event>0]

#create vector of composite survival estimate for unexposed group

s.unexp.composite <- s.events.composite[1:last.unexp.composite]

#create vector of composite survival estimate for exposed group

s.exp.composite <-

s.events.composite[first.exp.composite:total.length.composite]

#Lag the survival for the composite outcome to create a dataset with event

# times and survival at the previous event time

s.unexp.lag <- c(1,lag(s.unexp.composite))

s.unexp.lag <- head(s.unexp.lag,-1)

s.exp.lag <- c(1,lag(s.exp.composite))

s.exp.lag <- head(s.exp.lag,-1)

compdat.unexp<-data.frame(t.unexp.composite,s.unexp.composite,s.unexp.lag)

compdat.exp<-data.frame(t.exp.composite,s.exp.composite,s.exp.lag)

###########################################################################

## Step 4: Fit a second weighted KM curve for the cause-specific event of

## interest. The point is to use the survfit command to provide the

## weighted number of events and weighted risk set at each failure time

## point

AUCsurvival<-Surv(dat$start,dat$end,dat$haart)

AUCfit_adj<-survfit(AUCsurvival~idu,weights=s.ipw,data=dat,type="kaplan-meier")

###########################################################################

## Step 5: Find event times and survival at each event time for cause
## specific event of interest (same as Step 3)

t.events <- data.frame(AUCfit_adj$time[AUCfit_adj$n.event>0])

names(t.events)<-"t.events"

t.events$diff<-c(NA,diff(t.events$t.events))

last.unexp<-which(t.events$diff<0)-1

first.exp<-which(t.events$diff<0)

total.length<-length(t.events$t.events)

t.unexp <- t.events[1:last.unexp,1]

t.exp <- t.events[first.exp:total.length,1]

#Find event times for event of interest, by idu status and save in final

# dataset called "compfinal"

match.unexp<-compdat.unexp$t.unexp.composite%in%t.unexp

match.exp<-compdat.exp$t.exp.composite%in%t.exp

#save event times for event of interest among the unexposed in final dataset

compfinal.unexp<-compdat.unexp[which(match.unexp==T),]

#save event times for event of interest among the exposed in final dataset

compfinal.exp<-compdat.exp[which(match.exp==T),]

###########################################################################

## Step 6: Save the number at risk and number of events from running survfit ## command on the event of interest

#identify the weighted number at risk during each event time

risk.event <- AUCfit_adj$n.risk[AUCfit_adj$n.event>0]

#create vector of weighted number at risk at each event time for unexposed

# and exposed groups

risk.unexp<- risk.event[1:last.unexp]

risk.exp <- risk.event[first.exp:total.length]

#identify the weighted number of events at each event time

events.event <- AUCfit_adj$n.event[AUCfit_adj$n.event>0]

#create vector of weighted number of events at each event time for unexposed

# and exposed groups

events.unexp <- events.event[1:last.unexp]

events.exp <- events.event[first.exp:total.length]

#Add columns to final dataset: number at risk and number of events for event

# of interest

compfinal.unexp$risk.event<-risk.unexp

compfinal.unexp$events.event<-events.unexp

compfinal.exp$risk.event<-risk.exp

compfinal.exp$events.event<-events.exp

###########################################################################

## Step 7: Calculate the cumulative incidence function and survival function

## for the event of interest

CIF.unexp<-cumsum(compfinal.unexp$s.unexp.lag*

(compfinal.unexp$events.event/compfinal.unexp$risk.event))

CIF.exp<-cumsum(compfinal.exp$s.exp.lag*

(compfinal.exp$events.event/compfinal.exp$risk.event))

SF.unexp<-1-CIF.unexp

SF.exp<-1-CIF.exp

###########################################################################

## Step 8: Obtain values for Table 3: time to ART initiation treating death

## and loss-to-clinic as competing events

#set follow-up time to 5 years (1826.25 days)

stoptime<-1826.25

#Calculate the cause-specific RMST for unexposed group, accounting for

# competing events by iteratively adding the area under each step of the # survival function

area.unexp.cs <- NULL

for (i in

1:length(compfinal.unexp$events.event[compfinal.unexp$t.unexp<stoptime])){

area.unexp.cs[i] = SF.unexp[i-1]*(t.unexp[i]-t.unexp[i-1])

}

area.unexp.cs<-(sum(area.unexp.cs,na.rm=TRUE)+

SF.unexp[max(compfinal.unexp$t.unexp<stoptime)]*(stoptime-

max(compfinal.unexp$t.unexp[compfinal.unexp$t.unexp<stoptime])))

#Calculate the cause-specific RMST for exposed group, accounting for

# competing events by iteratively adding the area under each step of the # survival function

area.exp.cs <- NULL

for (i in 1:length(compfinal.exp$events.event[compfinal.exp$t.exp<stoptime])) {

area.exp.cs[i] = SF.exp[i-1]*(t.exp[i]-t.exp[i-1])

}

area.exp.cs<-sum(area.exp.cs,na.rm=TRUE)+

SF.exp[max(compfinal.exp$t.exp<stoptime)]*(stoptime-

max(compfinal.exp$t.exp[compfinal.exp$t.exp<stoptime]))

#Calculate the difference and ratio of RMST between exposed and unexposed

# groups for Table 3

AUC_diff.cs<-area.exp.cs-area.unexp.cs

AUC_ratio.cs<-area.exp.cs/area.unexp.cs

###########################################################################

###########################################################################

################

### TABLE 4 ####

################

# To obtain estimates in between the 1-CIF(t) of ART and CIF(t) of competing

# event, repeat steps for obtaining table 3 above replacing event of

# interest with competing event. Rather than calculate the area under the

# 1-CIF(t) of competing event, replace steps with calculating the area

# under CIF(t) of competing event. Then subtract this area for unexposed

# group from that obtained from the 1-CIF(t) of ART initiation to get

# area between 1-CIF(t) of ART and CIF(t) of competing event. Repeat

# for exposed group.
